# Urine Output Assessment in Acute Kidney Injury: The Cheapest and Most Impactful Biomarker

**DOI:** 10.3389/fped.2019.00565

**Published:** 2020-01-21

**Authors:** Stuart L. Goldstein

**Affiliations:** ^1^Center for Acute Care Nephrology, Cincinnati Children's Hospital Medical Center, Cincinnati, OH, United States; ^2^Department of Pediatrics, University of Cincinnati College of Medicine, Cincinnati, OH, United States

**Keywords:** acute kidney injury, oliguria, children, fluid overload, epidemiology

## Abstract

Acute kidney injury (AKI) is independently associated with morbidity and mortality in critically ill neonates, children, adolescents, and young adults. AKI occurs commonly in this population, and the vast majority of published studies utilize only a serum creatinine based criteria for AKI diagnosis and staging. While urine output criteria have been a part of all AKI systematic and multidimensional AKI definitions for the past 15 years, oliguria based on these definitions is difficult to extract from the electronic health record. This manuscript reviews the published data regarding the impact of oliguria on patient outcomes, and the contribution of oliguria to % fluid overload and resultant changes in serum creatinine based epidemiology. The aim of this manuscript is to demonstrate that oliguria is an incredibly valuable biomarker for the management of patients with, or at-risk for, AKI.

## Introduction

Acute kidney injury (AKI) occurs commonly in patients admitted to intensive care units and is independently associated with morbidity and mortality across the entire spectrum of patient age, from neonates to elderly adults (1–3). While extensive efforts have been focused on identifying markers of structural kidney injury (4), our current diagnostic AKI criteria still rely on changes in kidney function markers, namely serum creatinine, and urine output (5). Most studies to date have utilized only serum creatinine, as it is a discrete data field in the electronic health record with a singular validated measured value, whereas urine output requires a dynamic assessment of change with a time based value in the denominator. Recent data, however, have demonstrated that oliguria may have a stronger association with patient outcomes, and neglecting the urine output definition leads to missing a substantial proportion of patients with AKI.

The aims of this manuscript are to review the published data regarding the impact of oliguria on patient outcomes, and the contribution of oliguria to fluid overload and resultant changes in serum creatinine based epidemiology. After this review, it will become clear that oliguria is an invaluable biomarker in the management of the patient with, or at-risk for AKI.

## Impact of Oliguria on Outcomes in the Critically ILL Patient

Current diagnostic and staging criteria based on the recommendations of the Kidney Disease Improving Global Outcomes (KDIGO) Work Group for AKI are listed in Table 1 (5). Kellum et al. applied the serum creatinine and urine output AKI criteria in over 32,000 adults at their single center and assessed for associations with morbidity and mortality in patients who met the serum creatinine or urine output criteria alone and in combination (6). They observed a significant increase in hospital length of stay, renal replacement therapy provision and hospital, 30-, 90, and 1 year mortality in patients who experienced AKI by either criteria. Importantly, the occurrence of these poor outcomes nearly doubled in patients who developed AKI by both criteria vs. either criteria alone.

**Table 1:** AKI staging by the kidney disease: improving global outcomes disease (KDIGO) guidelines (5).

**AKI stage**	**Creatinine criteria**	**Urine output criteria**
Stage 1	1.5–1.9 x baseline OR ≥ 0.3 mg/dL absolute increase^*^	<0.5 mL/kg/h for 6–12 h
Stage 2	2.0–2.9 × baseline	<0.5 mg/kg/h for ≥12h
Stage 3	≥3x baseline, OR Increase in creatinine to 4 mg/dL, OR Decrease in eGFR to <35 mL/min/1.73 m^2^, OR Initiation of RRT^**^	<0.3 mL/kg/h for ≥24 h, OR Anuria for ≥12 h

* *While relative creatinine changes may occur over a 7 days period, this absolute change must occur over 48 h*.

** *Initiation of RRT was used as an outcome rather than a diagnostic criteria for this analysis*.

The multi-national prospective Assessment of Worldwide Acute kidney injury, Renal angina and Epidemiology (AWARE) study enrolled nearly 5,000 children to evaluate potential associations between KDIGO Stage 2 or 3 AKI and outcomes in critically ill children (2). Indeed, Stage 2 or 3 AKI was associated with a 1.77 increased risk of 28 days mortality, after controlling for 16 variables associated with mortality on univariable analysis. A recent study assessed the 3,318 patients in the AWARE cohort with sufficient serum creatinine and UOP data to compare outcomes based on AKI using either criterion alone, or in combination (7). Twenty-eight-day mortality was higher for patients Stage 2 or 3 AKI by creatinine criteria (6.7%) or UOP (7.8%) than patients with no AKI or Stage 1 AKI (2.9%). It is important to note that 18.1% of patients with Stage 2 or 3 AKI only met the UOP criteria, and would have been misclassified as not having severe AKI. While 28-day mortality did not differ between patients with Stage 2 or 3 AKI by creatinine alone vs. UOP alone, patients who met Stage 2 or 3 by both criteria experienced a 5-fold increased 28-day mortality rate (38.1%). Thus, taken together, these large adult and pediatric studies support routine assessment of both serum creatinine and UOP for AKI diagnosis in critically ill patients.

## Connection Between Oliguria and Resultant Fluid Overload and Effects on AKI Epidemiology

Multiple studies have demonstrated the association between ICU fluid accumulation and morbidity and mortality in children. These studies were the subject of a comprehensive systematic review and meta-analysis by Alobaidi et al. and an extensive review is beyond the scope of this paper (8). In general, however, fluid overload of >10–20% body weight was noted to be a threshold that demonstrated associations with prolonged mechanical ventilation, ICU length of stay and mortality, both in patients who received, or did not receive renal replacement therapy.

In the following case, we can assess the resultant fluid accumulation status based on a standard volume of fluid resuscitation, required daily intake, and oliguria based on KDIGO staging. The case involves a 30 kg (body surface area of 1 m^2^) patient with septic shock. We will assume the patient receives 60 ml/kg of crystalloid (1,800 ml) for fluid resuscitation and then is put on a fixed rate of fluid of 1,600 ml/day (based on a standard rate of 1,600 ml/m^2^ BSA for a patient with normal kidney and other homeostatic functions). Table 2 details the percent fluid accumulation per unit time as defined by the upper limit of UOP for each of the three KDIGO AKI strata. These calculations demonstrate that the patient will achieve the 10% fluid overload threshold within 24 h by KDIGO Stage 2 or 3 criteria, and would be more than halfway to 10% fluid overload at 6–12 h with by Stage 1 criteria. Thus, it is extremely important to note the UOP rate and duration early in the ICU course, as the patient is at risk of developing significant fluid accumulation association with morbidity and mortality.

**Table 2:** How do the KDIGO UOP AKI criteria impact fluid accumulation in a case of septic shock after 24 hours?

**KDIGO stage**	**Metric**	**Duration**	**%FO**
1	<0.5 ml/kg/h for 6–12 h	6 h (1,800 + 400 ml In−90 ml Out)	7.0
		12 h (1,800 + 800 ml In−180 ml Out)	8.1
2	<0.5 ml/kg/h ≥12 h	24 h (1,800 + 1,600 ml In−360 ml Out)	10.1
3	<0.3 ml/kg/h for 24 h	24 h (1,800 + 1,600 ml In−216 ml Out)	10.6
	OR	12 h (1,800 + 800 ml In−0 ml Out)	8.7
	Anuria	24 h (1,800 +1,600 ml In−0 ml Out)	11.3

Assumptions for calculations:

*1) 30 kg patient (body surface area = 1.0 m^2^) with septic shock*.

*2) 1,800 ml fluid resuscitation (60 ml/kg)*.

*3) 1,600 ml/day standard fluid prescription (BSA metric)*.

*4) Upper limit of KDIGO Urine Output criteria*.

*5) Insensible losses not factored into the calculation since critically ill patients are often intubated and have minimal insensible losses*.

As noted in the previous section, nearly 20% children in the AWARE study who met the KDIGO UOP AKI criteria did not meet the serum creatinine criteria. Recent studies have demonstrated the impact of fluid accumulation on AKI ascertainment by serum creatinine criteria, with the concept that serum creatinine may be diluted in a fluid overloaded patient, thereby blunting the creatinine rise and leading to a false negative assessment for AKI. Liu et al. performed this assessment in the Fluid And Catheter Treatment Trial (FACTT), which randomized 1,000 adult patients to a liberal vs. conservative fluid provision strategy after resuscitation (9). They found a significant reclassification of AKI in the patients randomized to the liberal fluid administration group, and a higher mortality rate in patients reclassified from not having AKI to having AKI based on creatinine correction for fluid overload, compared to patients who did not have AKI after creatinine correction. Basu et al. performed a similar exercise in a pediatric cohort undergoing the arterial switch operation for transposition of the great vessels (10). In this study, AKI was associated with poor outcomes including higher postoperative day 1 fluid balance, higher inotrope scores postoperative days 1 and 2, and longer: postoperative ICU length of stay, overall ICU length of stay, and postoperative hospital length of stay. All of these associations between AKI and morbidity strengthened after AKI status was reclassified based on serum creatinine correction for fluid overload. Table 3 illustrates the impact of KDIGO UOP criteria on fluid balance and serum creatinine dilution applied to the case of the 30 kg patient with septic shock, assuming a measured serum creatinine of 1.0 mg/dl, after 12–24 h, the dilutional effect led to a 10–16% decrease in serum creatinine levels.

**Table 3:** How do the KDIGO UOP criteria affect serum creatinine based on a dilutional effect after 24 hours?

**KDIGO stage**	**Metric**	**Duration**	**+FB (ml)**	**Corr [SCr]**
1	<0.5 ml/kg/h for 6–12 h	6 h (1,800 + 400 ml In−90 ml Out)	2,100	0.90
		12 h (1,800 + 800 ml In−180 ml Out)	2,420	0.88
2	<0.5 ml/kg/h > 12 h	24 h (1,800 + 1,600 ml In−360 ml Out)	3,040	0.86
3	<0.3 ml/kg/h for 24 h	24 h (1,800 +1,600 ml In−216 ml Out)	3,184	0.85
	OR	12 h (1,800 + 800 ml In−0 ml Out)	2,600	0.87
	Anuria	24 h (1,800 +1,600 ml In−0 ml Out)	3400	0.84

Assumptions for calculations:

*1) 30 kg patient with septic shock*.

*2) 1,800 ml fluid resuscitation (60 ml/kg)*.

*3) 1,600 ml/day standard fluid prescription (BSA metric)*.

*4) Upper limit of KDIGO Urine Output*.

*5) Volume of distribution of water = 18,000 ml (600 ml/kg)*.

*6) Corrected [SCr] = [(1 mg/dl) × 18,000 ml/(18,000 ml) + fluid balance (FB)]*.

*7) Insensible losses not factored into the calculation since critically ill patients are often intubated and have minimal insensible losses*.

## Conclusions

In summary, most published studies in the AKI field rely on serum creatinine based definitions, due to their ease of data extraction from the electronic health record as well as the technical challenges of urine collection in neonates, infants and toddlers without an indwelling bladder catheter (11). Yet, AKI defined by oliguria often portends a worse prognosis and the AKI diagnosis would be missed in a substantial proportion of patients where UOP is not assessed. While we don't yet understand the mechanisms for the worse outcomes with oliguria, all of these observations support the concept that assessment and recognition of oliguria, and its effect on patient fluid accumulation and serum creatinine based AKI diagnosis ascertainment, are crucial for management of critically ill patients at risk for AKI. It is important to note however, that early identification of patients at risk for severe AKI and adverse outcome can influence physician's decision-making, e.g., with regard to the implementation of the critical care bundle for AKI or with regard to the early insertion of catheters for subsequent initiation of renal replacement therapy. Hence, therapeutic management of AKI benefits from criteria that allow for an immediate diagnosis of AKI, integrating the information regarding the impact of UOP on AKI diagnosis presented in this article.

## Author Contributions

SG wrote the article and is responsible for all of the content.

### Conflict of Interest

The author declares that the research was conducted in the absence of any commercial or financial relationships that could be construed as a potential conflict of interest.
